# Developmental vitamin D deficiency increases foetal exposure to testosterone

**DOI:** 10.1186/s13229-020-00399-2

**Published:** 2020-12-10

**Authors:** Asad Amanat Ali, Xiaoying Cui, Renata Aparecida Nedel Pertile, Xiang Li, Gregory Medley, Suzanne Adele Alexander, Andrew J. O. Whitehouse, John Joseph McGrath, Darryl Walter Eyles

**Affiliations:** 1 Queensland Brain Institute, The University of Queensland, St Lucia, QLD Australia; 2 Queensland Centre for Mental Health Research, The Park Centre for Mental Health, Wacol, QLD Australia; 3 Telethon Kids Institute, The University of Western Australia, Perth, WA 6009 Australia; 4 NCRR—National Centre for Register-Based Research, Department of Economics and Business Economics, Aarhus University, Aarhus C, Denmark

**Keywords:** Developmental vitamin D deficiency, Autism, Testosterone, Aromatase, Methylation, Animal model

## Abstract

**Background:**

Autism spectrum disorder (ASD) is a group of neurodevelopmental disorders which are more common in males. The ‘prenatal sex steroid’ hypothesis links excessive sex-steroid exposure during foetal life with the behavioural differences observed in ASD. However, the reason why sex steroid exposure may be excessive remains unclear. Epidemiological studies have identified several environmental risk factors associated with ASD, including developmental vitamin D (DVD) deficiency. We have demonstrated in an animal model that DVD-deficiency is associated with a hyper-inflammatory response in placentas from male but not female foetuses. Vitamin D also regulates the expression of several steroidogenic enzymes in vitro. Therefore using this animal model, we have examined whether DVD-deficiency leads to increased sex-steroid levels in both the maternal and foetal compartments.

**Methods:**

Female rats are fed a vitamin D deficient diet from 6 weeks before mating until tissue collection at embryonic day 18. We examined the levels of testosterone, androstenedione and corticosterone in maternal plasma, foetal brains and amniotic fluid. We further examined gene expressions of steroidogenic enzymes and DNA methylation of aromatase promoters in foetal brains as a potential molecular mechanism regulating testosterone expression.

**Results:**

We show that DVD-deficiency increases testosterone levels in maternal blood. We also show elevated levels of testosterone and androstenedione in the amniotic fluid of female but not male DVD-deficient foetuses. Testosterone levels were also elevated in DVD-deficient male brains. Vitamin D, like other steroid-related hormones, regulates gene expression via methylation. Therefore we examined whether the significant elevation in testosterone in male brains was due to such a potential gene-silencing mechanism. We show that the promoter of aromatase was hyper-methylated compared to male controls.

**Limitations:**

A reduction in aromatase, in addition to causing excessive testosterone, could also lead to a reduction in estradiol which was not examined here.

**Conclusions:**

This study is the first to show how an epidemiologically established environmental risk factor for ASD may selectively elevate testosterone in male embryonic brains. These findings provide further mechanistic support for the prenatal sex steroid theory of ASD.

## Background

Vitamin D has long been known to regulate calcium homeostasis and promote healthy bones [1]. Recent clinical and pre-clinical studies have informed the research community on a much broader role of vitamin D. For example, in addition to its role in calcium absorption, vitamin D affects fundamental developmental processes such as cellular differentiation and regulation of immune function [2, 3]. Evidence also continues to accumulate, suggesting that vitamin D is an active neurosteroid and developmental vitamin D (DVD) deficiency is associated with adverse brain outcomes [4]. Two recent epidemiological studies have linked low maternal vitamin D levels during pregnancy and an increase in the risk of autism spectrum disorder (ASD) diagnosis in offspring. The first of these studies showed that vitamin D deficiency from mid-gestation to birth is associated with ASD-related traits in a large Dutch population-based cohort [5]. This study also assessed ASD diagnosis in children and confirmed this relationship [6]. The second study was a large case–control cohort from Sweden. This study found that both maternal and neonatal vitamin D deficiencies are associated with increased risk of developing ASD [6]. This relationship has also been replicated by two other studies [7, 8]. It is important to note two recent studies did not support this link [9, 10]. However the populations in which these two studies were conducted had far higher mean levels of vitamin D > 75 nM which is regarded as vitamin D sufficient [11], thus minimising the possibility of testing this relationship. In addition to this, DVD-deficient animals display several behavioural traits relevant to the core ASD symptoms across a range of developmental stages such as delays in motor development, altered ultrasonic vocalizations, stereotyped repetitive behaviour and deficits in social play behaviour [12, 13]. Some behaviours such as delays in motor development were more prominent in males.

Vitamin D also plays an important role in the prevention of ASD. For example, there is one study conducted to investigate the effect of maternal vitamin D supplementation on the reoccurrence of ASD in “high risk” group [14]. This open-label prospective study showed that expectant mothers who already had at least one autistic child, when supplemented with high doses of vitamin during subsequent pregnancy (5000 IU/day), and then gave 1000 IU/day to their newborns for 3 years, had offspring with a reduced reoccurrence of ASD. This study while promising unfortunately had a small sample size and no control group so can therefore be considered as a preliminary model for future clinical trials into vitamin D supplementation at best. Furthermore, an animal study shows that vitamin D supplements during pregnancy in an animal model of maternal immune activation also completely prevent ASD traits in their offspring [15].

ASD is a neurodevelopmental disorder characterized by early deficiencies in social interaction and communication together with highly repetitive stereotyped behaviour [16]. While ASD is known to involve a genetic component, the biological pathways contributing to ASD likely vary between individuals [17]. ASD has a pronounced gender-biased prevalence with an estimated male to female diagnostic ratio of 3:1 [18]. Increased exposure of the male foetus to testosterone has been identified as one plausible reason for such sex differences. More direct support comes from amniocentesis studies. Two studies from the same group have shown elevated levels of testosterone in the amniotic fluid of children who later went on to develop ASD [19–21]. Androstenedione which is a weak androgen and precursor for testosterone was also found to be elevated in the amniotic fluid of ASD children [19]. Direct correlations between foetal testosterone and ASD traits have also been demonstrated in many studies [20, 22, 23].

**Fig. 1:**
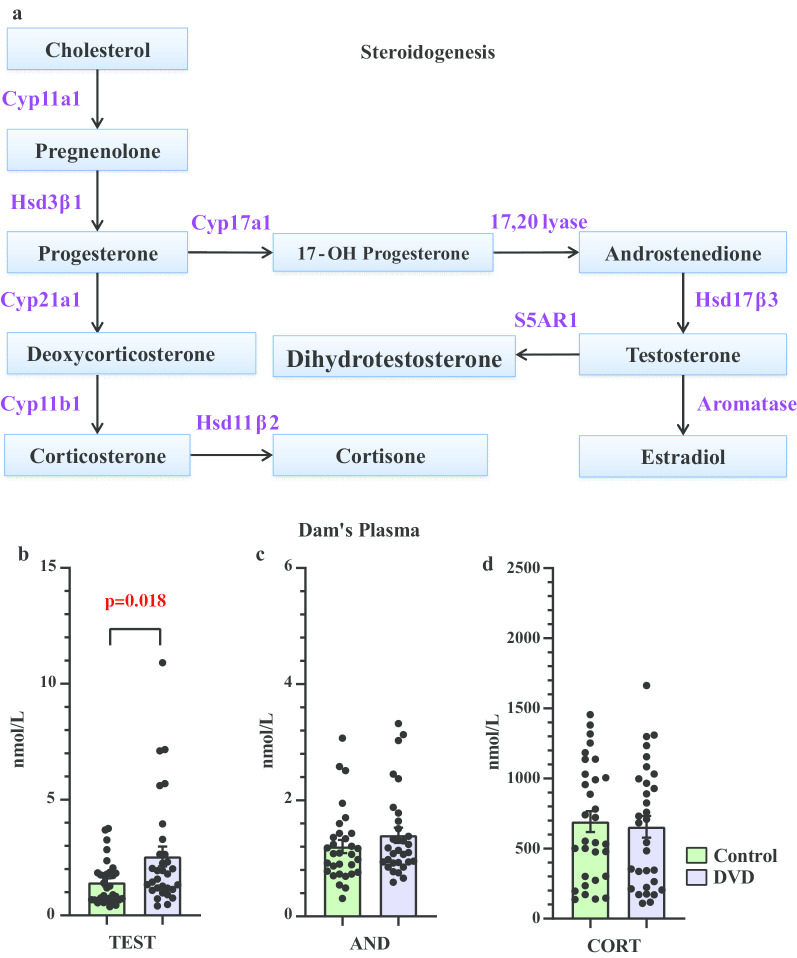
**a** Schematic representation of steroid hormone biosynthesis. All listed enzymes have been shown to be regulated by vitamin D, **b** steroid levels in dam’s plasma. Transient vitamin D deficiency in pregnant rats significantly increased plasma testosterone levels but not **c** androstenedione or **d** corticosterone *n* = 31 control, *n* = 31 DVD. *TEST* testosterone, *AND* androstenedione, *CORT* corticosterone. Error bars show SEM (*p* < 0.05)

Vitamin D (25OHD) is converted into its active form 1,25-dihydroxy-vitamin D (1,25OHD) by the enzyme *CYP27B1*. Like other steroids, 1,25OHD regulates gene expression through its nuclear receptor—the vitamin D receptor [24]. Vitamin D plays an important role in modulating steroidogenesis by regulating the expression of several genes encoding steroidogenic enzymes and of most relevance to this study, *CYP19A1* the gene coding for aromatase which is the major catabolic enzyme in testosterone elimination (see Fig. 1a) [25]. Vitamin D has also been inversely correlated with testosterone in numerous tissues and cell types [26, 27]. Taken together, this evidence suggests that DVD-deficiency might contribute to higher testosterone levels in different tissues.

The primary aim of this project was to examine if DVD-deficiency increases foetal exposure to sex-steroids. Here we have examined testosterone, androstenedione and corticosterone in maternal blood, foetal brain and amniotic fluid. We selected these steroids because they are elevated in ASD and regulated by vitamin D [28–30]. We also examined the level of 2-Methoxyestradiol (2-ME) from the maternal serum. 2-ME is a natural metabolite of estradiol and implicated in preeclampsia which is a known risk factor for ASD [31–33]. We further examined the expression of multiple cytochrome P450 enzymes and hydroxysteroid dehydrogenases responsible for sex-steroids formation or elimination. Finally, we explored plausible vitamin D-related molecular mechanisms for alterations in enzyme expression.

## Methods

### Tissue collection

DVD-deficiency was induced by feeding standard casein AIN93G rodent chow (Speciality Feeds, Western Australia) without added vitamin D (0 IU cholecalciferol, Product # SF09-105) to 4-week-old female Sprague–Dawley (SD) rats (Animal Resource Centre, Western Australia). Control female rats were fed control casein AIN93G diet (Product # SF09-104) containing 1000 IU of cholecalciferol. Both control and vitamin D deficient female rats were time-mated with vitamin D normal SD sires at 10 weeks of age. For time-mating, four females were housed with one male in a single cage and the vaginal plug was checked every 24 h in the morning. Successful mating was verified by the presence of a vaginal plug, and the day was referred to as embryonic day (E) 0. Pregnant dams were euthanized at E18 which represents the peak foetal period of steroidogenesis in SD rats [34]. Maternal blood, foetal brains and amniotic fluid were collected. For detailed tissue collection procedures and further information regarding animal cohorts used in the study see Additional file 1.

### Steroid assay

Foetal brains were homogenised in 1.0 mL cold 1:1 acetone:ethanol using an ultrasonic homogeniser (Vibra-Cell, Sonic & Materials Inc Newtown, CT). Brain homogenates were centrifuged and the supernatant was removed and 200 μL of internal standard (1.2 nM testosterone-[^2^H_5_], 2.5 nM 4-androstene-3,17-dione-[^13^C_3_] in methanol and 125 nM corticosterone-[^2^H_4_] in acetonitrile) was added to each sample and diluted with 3.0 mL of milliQ water. Diluted samples were loaded onto conditioned Strata C18-E (50 mg/mL SPE cartridges (Phenomenex)). Samples were eluted with 1.0 mL of 100% methanol and evaporated to dryness in the vacuum centrifuge (miVac Sample Concentrators SP Scientific Warminster, PA) at approximately 40 °C for 60 min and stored at − 20 °C. For blood samples, 1.0 mL cold 1:1 acetone:ethanol was added to 20 µL plasma which was then processed similar to foetal brains.

For amniotic fluid, 50 µL of sample and standard were dispensed into a single well of a 96 deep-well plate. After that 200 μL of internal standard (1.2 nM testosterone-[^2^H_5_], 2.5 nM 4-androstene-3,17-dione-[^13^C_3_] in methanol and 125 nM corticosterone-[^2^H_4_] in acetonitrile) was added to each well, vortexed after which samples were transferred to an already conditioned 96 well Hydrophilic-Lipophilic-Balanced, water-wettable, reversed-phase sorbent plate (Oasis HLB Waters Corporation MA USA). All eluates were evaporated to dryness and stored at − 20 °C. Steroid levels were quantified by an in-house Liquid Chromatography/ Tandem Mass Spectrometry (LC–MS/MS) technique. The system consisted of a Shimadzu Nexera^®^ UPLC system with a Phenomenex Kinetex^®^ 1.7u XB-C_18_ 100 Å (50 × 2.1 mm) column attached to an AB Sciex QTrap-5500^®^ triple-quadrupole mass spectrometer. On the day of assessment dried samples were reconstituted in 150 μL of 1:1 methanol:water and 40 µL of sample extract (foetal brain, dam’s blood and amniotic fluid) was injected onto a UPLC system and eluted at 500 µL/min using a gradient method with mobile phase *A* = 0.1% aqueous formic acid and mobile phase *B* = 0.1% formic acid in 95:5 acetonitrile:water. The gradient began at 0% mobile phase *B* and increased to 76% mobile phase *B* at 4 min and then to 95% mobile phase *B* for 2 min. The detection on an API5500-QTrap (Applied Biosystems) was performed using positive ion MRM mode with electrospray ionization. The mass-spectrometer settings for each mass transition for the examined steroids and their internal standards are as follows: for corticosterone m/z = 347.1 → 329.1, declustering potential, (DP) = 120 V, collision energy (CE) = 23 V; for corticosterone—[^2^H_4_] 351 → 333 DP = 120 CE = 18; for testosterone 289.1 → 97 DP = 130 CE = 29; for testosterone-[^2^H_5_] 294 → 100 DP = 75 CE = 29; for 4- androstenedione 287 → 97 DP = 136 CE = 31; for]; 4-androstene-3,17-dione-[^13^C_3_] 290 → 100 DP = 145 CE = 29. Calibration standards and three levels of quality controls were prepared in protein stripped urine (Mass Spec Gold Urine, Golden West Biologicals). Steroid quantifications were performed using MultiQuant™ software version 2.1 (AB SCIEX, MA, Framingham, USA) by isotope dilution and comparison with a standard curve. The calibration range was 0.05–3.2 nM for testosterone, 0.2–12.8 nM for androstenedione and 10–640 nM for corticosterone. Test samples were diluted where necessary to be within range.

### Expression of steroid metabolizing enzymes

Expressions of genes encoding steroidogenic enzymes known to be regulated by vitamin D were assessed using qPCR. See Additional file 1 for qPCR conditions and a list of primers used in this study.

### Methylated DNA immunoprecipitation

The levels of DNA methylation within the promoter of genes encoding aromatase were measured by methylated DNA immunoprecipitation (MeDIP) followed by qPCR. Briefly, DNA was extracted from embryonic brains (4 animals per diet group) using Qiagen DNeasy Blood and Tissue extraction kit following the manufacturer’s instructions. MeDIP was performed as previously described by Hu et al. [35]. Briefly, the isolated genomic DNA was digested with RNase A, randomly fragmented by ultra-sonication (Covaris) into fragments of approximately 500 bp in length, and denatured at 95 °C for 10 min within denature buffer and put it on ice right away. A total of 2 µg of fragmented DNA were used for each MeDIP assay. 10% of fragmented DNA from each sample was saved and kept as input genomic DNA. The remaining sonicated DNA was diluted in 300 µL in IP buffer (10 mM sodium phosphate, 140 mM NaCl, and 0.05% triton X-100). Overnight incubation with mouse anti-5-methylcytosine (5mC, Abcam) antibody or IgG antibody as non-specific control (Abcam) was performed to generate IP enriched fragments. After overnight incubation, samples were incubated with magnetic beads (Dynabeads G) for 2 h, washed and treated with proteinase K for 3 h at 55 °C. The immunoprecipitated DNA was extracted using phenol–chloroform followed by ethanol precipitation.

The qPCR reaction was performed in LightCycler^®^ 480 System (Roche Diagnostics, Penzberg, Germany) using SensiFAST SYBR Green Master Mix (Bioline Meridian Bioscience, Memphis, TN, USA) under the following conditions: initial denaturation at 95 °C for 10 min followed by 40 cycles of amplification (95 °C for 15 s, then 60 °C for 20 s, then 72 °C for 30 s). We performed qPCR on input (1/10) and immunoprecipitated fragments for each sample, using primers spanning the − 2000 bp of the 2 aromatase promoters (PII and PI.f). Primers were designed by using a 500 bp sliding window to identify areas where the methylation levels of the proximal promoter changed when compared to control (Additional file 1: Table S2). Relative 5mC enrichment was calculated as a percentage of input genomic DNA.

### Statistical analysis

Results were analysed using IBM SPSS (version 25.0) Armonk, NY, USA. Steroid data from maternal plasma were analysed by independent *t*-test. Steroids levels in foetal brain and amniotic fluid were analysed by multivariate analysis of variance to determine the main effect of foetal sex, maternal diet and foetal sex × diet interactions. Testosterone levels in the female amniotic fluid were frequently below the limit of assay methods so were analysed as categorical values rather than a continuous measure by a Chi-squared test. Expressions of steroid metabolizing enzymes were first analysed by repeated measure ANOVA. Significant effects were then followed by independent *t*-tests. When multiple *t*-tests were conducted, a Benjamini–Hochberg correction was performed to account for multiple comparisons. Relative gene expression was calculated by the comparative *C*_T_ method [36]. Significantly altered genes from a 1st run are repeated (technical replicates) in a completely new second run to avoid reporting false-positive findings. Only genes in which there is a significant alteration in both runs are reported as significantly changed. MeDIP-qPCR data were analysed by paired *t*-test. The effect size for mean differences of groups with equal sample size was calculated by Cohen’s d, and groups with unequal sample size were calculated by Hedges' g. The level of statistical significance was defined as *p* < 0.05. We expect dietary intervention will produce a small effect size on steroid levels; hence a sufficiently large sample size was employed.

## Results

### Steroid levels in the dams

The circulating levels of steroids were assessed in maternal plasma. In vitamin D deficient dams there was an increase in plasma testosterone levels (*t*_(60)_ = 2.46, *p* = 0.018, Cohen’s *d* = 0.61) (Fig. 1b). However, androstenedione (*t*_(60)_ = 1.13, *p* = 0.26, Cohen’s *d* = 0.28) and corticosterone (*t*_(60)_ = 0.34, *p* = 0.73, Cohen’s *d* = 0.087) levels were not significantly changed by diet (Fig. 1c, d). The 2-ME levels were also not significantly different (*t*_(25)_ = 0.79, *p* = 0.38, Cohen’s *d* = 0.35) between DVD-deficient and control dams. Please see Additional file 1 for more details.

### Steroid concentrations in foetal brains

**Fig. 2:**
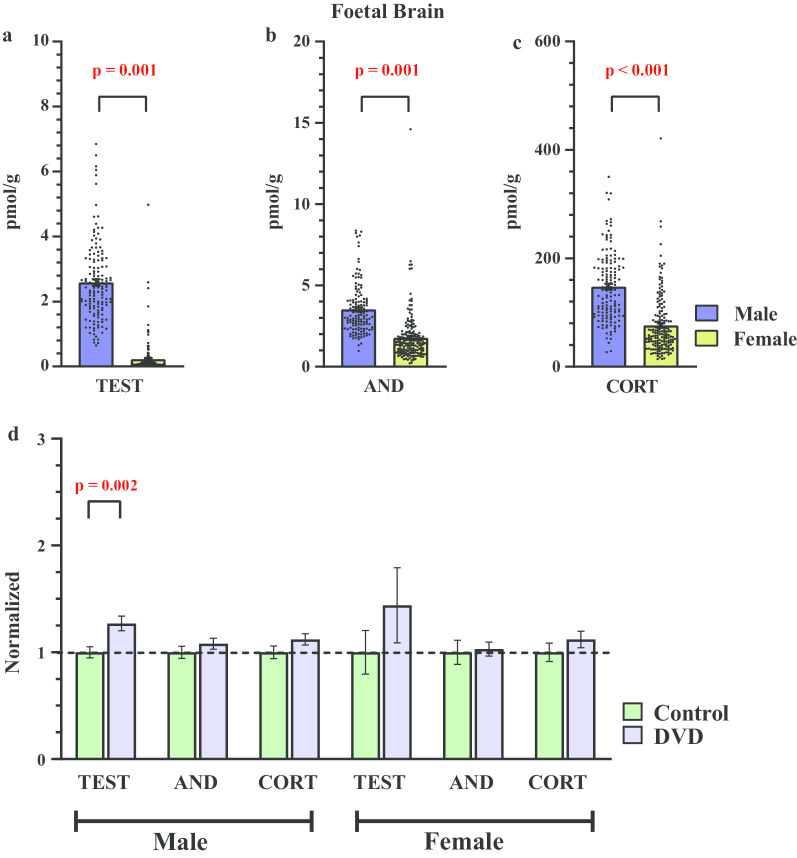
Effect of foetus sex and DVD-deficiency on steroid levels in foetal brain. Male foetal brains had significantly higher levels of **a** testosterone, **b** androstenedione, **c** corticosterone measured as pmol/g of brain tissue. *n* = 141 males, *n* = 177 females. **d** DVD-deficiency increased testosterone synthesis in male and female foetal brains, but this was only significant in males. Levels of androstenedione and corticosterone were not affected by maternal diet. Data is normalized to control group. *n* = 64 control males, *n* = 77 DVD-deficient males, *n* = 86 control females, *n* = 91 DVD-deficient females. *TEST* testosterone, *AND* androstenedione, *CORT* corticosterone. Error bars show SEM

As expected, there was a main effect of sex on testosterone levels in foetal brains. Testosterone levels were significantly higher in male foetal brains (*F*_1, 318_ = 565.7, *p* = 0.001, Hedges' *g* = 2.65) than the females (Fig. 2a). More importantly there was also a significant main effect of maternal diet on testosterone levels in foetal brains. Testosterone levels were significantly elevated (*F*_1, 318_ = 12.6, *p* = 0.001, Hedges' *g* = 0.26) in DVD-deficient brains compared with controls brains (Fig. 2d). There was also a sex × diet interaction (*F*_1, 318_ = 7.46, *p* = 0.007) on testosterone levels in the brains. Post hoc analysis revealed that this dietary effect was mainly driven by male (*t*_(139)_ = 3.19, *p* = 0.002, Hedges' *g* = 0.52) and not female foetal brains (*t*_(175)_ = 1.07, *p* = 0.29, Hedges' *g* = 0.16).

Again, as expected foetal brain androstenedione levels were significantly (*F*_1, 318_ = 101.7, *p* = 0.001, Hedges' *g* = 1.15) higher in the males compared to females foetuses (Fig. 2b). However, there was no main effect of maternal diet (*F*_1, 318_ = 0.85, *p* = 0.35, Hedges' *g* = 0.11) or foetal sex × diet interaction (*F*_1, 318_ = 0.36, *p* = 0.54) on androstenedione levels in foetal brains (Fig. 2d).

Corticosterone levels were also significantly higher in male foetal brains (*F*_1, 318_ = 111.8, *p* = 0.001, Hedges' *g* = 1.20) (Fig. 2c). However, there was no main effect of maternal diet (*F*_1, 318_ = 3.63, *p* = 0.057, Hedges' *g* = 0.21) nor was any foetal sex × maternal diet interaction (*F*_1, 308_ = 0.35, *p* = 0.55) on corticosterone levels in foetal brains (Fig. 2d).

### Expression of key the steroidogenic enzyme in foetal male brains

**Fig. 3:**
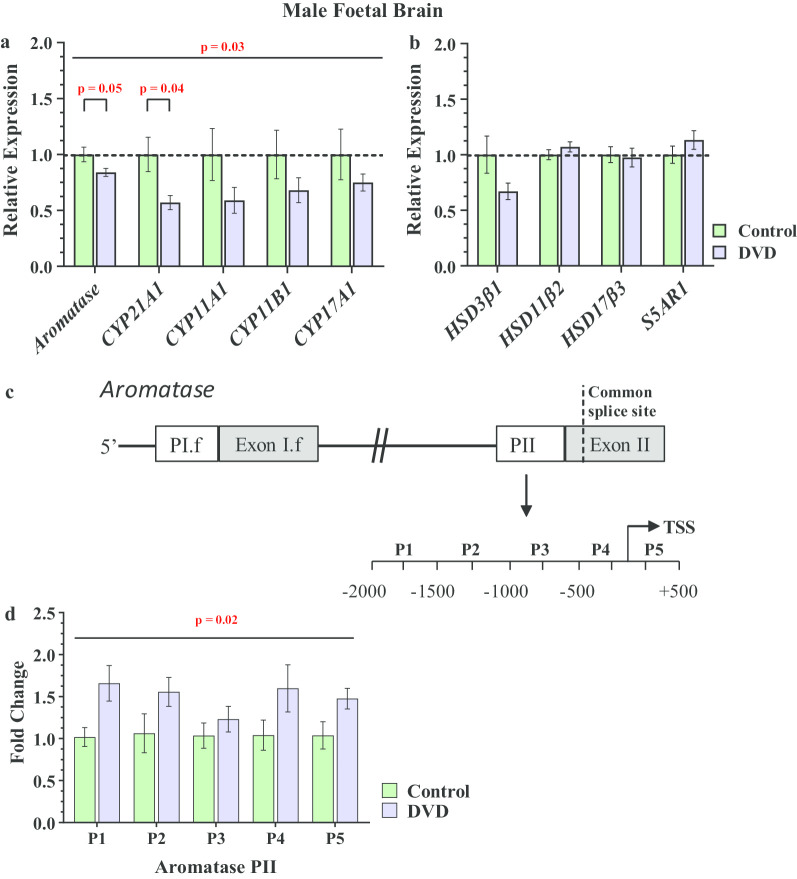
Expression of steroidogenic genes and comparative methylation of aromatase promoters in male foetal brains. **a** As a group, DVD-deficiency decreased cytochrome P450 but not the **b** hydroxyl-steroid dehydrogenases and steroid 5 Alpha-Reductase 1 (S5AR1). Of the individual cytochrome P450s enzymes, aromatase and *CYP21A1* were significantly down-regulated in DVD-deficient foetal male brains compared to controls. Data expressed as relative change to control levels. *n* = 24 control males, *n* = 21 DVD males. **c** Diagram depicting the 2 putative promoters of aromatase in the rat brain. Primers were designed for both promoters using a 500 bp sliding window relative to the transcription start site (TSS) in order to identify areas where the methylation levels of the proximal promoter changed when compared to control. **d** 5 mC levels were significantly higher within aromatase PII promoter in DVD-deficient male brains. *n* = 4 control males, *n* = 4 DVD males. Error bars show means SEM (*p* < 0.05)

Since there were no significant effects of maternal diet on any of the examined steroids in female brains, only male brains were subjected to any further analysis. Repeated measure ANOVA revealed that as a group, all cytochrome P450 enzymes were reduced in DVD-deficient male foetal brains (*F*_1, 43_ = 4.80 *p* = 0.03). However, when these data were further analysed by independent *t*-tests, only aromatase (*CYP19A1*) and *CYP21A1* were significantly changed. Consistent with the increase in testosterone in foetal male brains, aromatase, the major catabolic enzyme for testosterone was significantly reduced by DVD-deficiency in male foetal brains (*t*_(43)_ = 2.01, *p* = 0.05, Hedges' *g* = 0.63) (Fig. 3a). We next examined aromatase promoter methylation as a plausible mechanism for this reduction. MeDIP-qPCR was used to determine the 5 methylated Cytosine (5 mC) content of the aromatase promoter. In the rat brain, aromatase expression is driven by two different promoters PII and PI.f [37, 38]. Therefore, in this study we analysed the methylation status of the − 2000 bp proximal region of PII and PI.f promoters in foetal brains (Fig. 3c). Our result showed that 5mC levels were significantly increased (*t*_(5)_ = 2.53, *p* = 0.02, Cohen’s *d* = 0.60) along the PII promoter of aromatase in DVD-deficient brains when compared with controls (Fig. 3d). However, no significant differences was observed for the PI.f promoter (*t*_(7)_ = 1.65, *p* = 0.14).

Finally, as a group the expression of the hydroxyl steroid dehydrogenases in foetal male brains was shown to be unaffected by maternal diet (*F*_1, 43_ = 1.00, *p* = 0.32). In particular *HSD17β3* a major enzyme in testosterone formation was unaltered by DVD-deficiency (Fig. 3b). Therefore, it is likely that reduced elimination rather than increased formation is responsible for the increased levels of testosterone in male DVD-deficient brains.

*CYP21A1* is another gene which was significantly (*t*_(43)_ = 4.31, *p* = 0.04, Hedges' *g* = 0.63) reduced in male DVD-deficient foetal brains. *CYP21A1* is mainly involved in the biological conversion of deoxycorticosterone from progesterone. As corticosterone levels were not affected by maternal diet and progesterone was not measured, we did not pursue any mechanism for this apparent reduction in this study.

### Steroid concentrations in amniotic fluid

**Fig. 4:**
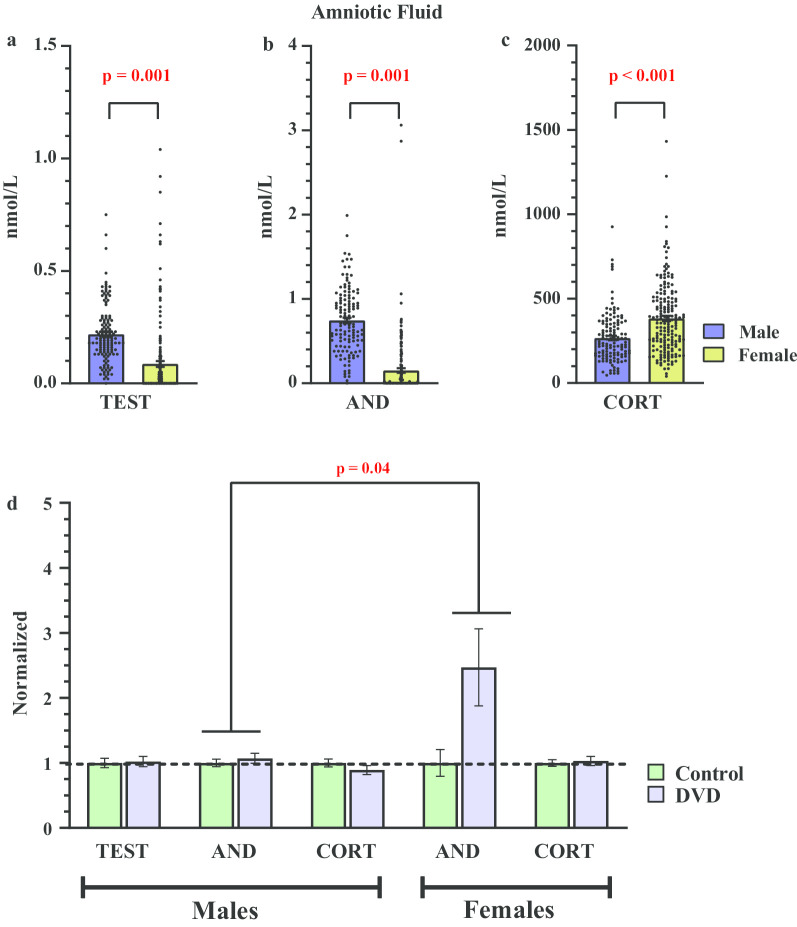
Effect of foetal sex and DVD-deficiency on steroid levels in amniotic fluid. Male amniotic fluid had significantly higher levels of **a** testosterone and **b** androstenedione compared to females. **c** However, corticosterone levels were higher in female amniotic fluid. *n* = 128 males, *n* = 180 females. **d** Androstenedione levels were significantly elevated by DVD-deficiency in the amniotic fluid of both male and female foetuses. Data is normalized to control group. *n* = 74 control males, *n* = 54 DVD males, *n* = 92 control females, *n* = 88 DVD females. *TEST* testosterone, *AND* androstenedione, *CORT* corticosterone. Error bars show SEM

As expected, there was a main effect of foetal sex on amniotic fluid testosterone. Testosterone levels were significantly higher in males compared with female amniotic fluid (*F*_1, 308_ = 52.4, *p* = 0.001, Hedges' *g* = 0.82) (Fig. 4a). Similar to foetal brains, there was also a main effect of maternal diet on amniotic fluid testosterone. Levels were significantly elevated in the amniotic fluid collected from DVD-deficient foetuses compared with controls (*F*_1, 308_ = 7.15, *p* = 0.008, Hedges' *g* = 0.28) (Fig. 4d). There was also a significant interaction of foetal sex × maternal diet (*F*_1, 308_ = 5.76, *p* = 0.017) on amniotic fluid testosterone. A further post hoc analysis revealed that opposite to foetal brains, in amniotic fluid this dietary effect was mainly observed in females but not males. Student *t*-test showed that DVD-deficiency does not affect testosterone levels in the male amniotic fluid [*t*(126) = 0.21, *p* = 0.83, Hedges' *g* = 0.03]. However, testosterone levels in many of the control female amniotic fluid samples were undetectable leading to a highly skewed distribution. Therefore, we performed Pearson Chi-square test. There was a significant association between the maternal diet and testosterone levels in the female amniotic fluid (*Χ*^2^(1) = 6.59, *p* < 0.008). Chi-squared tests revealed that DVD-deficient females were more likely to have measurable testosterone levels compared with control females (see Table 1).

**Table 1:** Chi-square tests of independence in the female amniotic fluid for testosterone

Female amniotic fluid testosterone	Detectable levels	Undetectable levels	Sample size	Chi-square tests of independence
	n	n	N	
Control	41	51	92	*X*^2^ (1) = 6.59
DVD-deficient	56	32	88	*p* < 0.008

In female amniotic fluid, testosterone levels were very low with many 0 values in the control sample. Therefore data were analysed non-continuously via Chi-squared test. *n* = 92 control females, *n* = 88 DVD females

Again, there was a significant main effect of foetal sex on androstenedione levels in amniotic fluid. Male amniotic fluid had significantly higher levels of androstenedione than females (*F*_1, 308_ = 188.8, *p* = 0.001, Hedges' *g* = 1.58) (Fig. 4b). Amniotic fluid androstenedione levels were also affected by maternal diet. DVD-deficient foetuses had significantly (*F*_1, 308_ = 4.21, *p* = 0.04, Hedges' *g* = 0.12) higher levels of androstenedione in the amniotic fluid compared with controls (Fig. 4d). However, there was no foetal sex and maternal diet interaction on amniotic fluid androstenedione levels (*F*_1, 308_ = 0.56, *p* = 0.37).

Corticosterone levels in the amniotic fluid were also affected by foetal sex. In contrast to both testosterone and androstenedione, corticosterone levels were significantly lower in male amniotic fluid compared to females (*F*_1, 308_ = 29.0, *p* = 0.001, Hedges' *g* = 0.61) (Fig. 4c). There was however no significant main effect of maternal diet (*F*_1, 308_ = 0.20, *p* = 0.66, Hedges' *g* = 0.009) or foetal sex × maternal diet interaction on corticosterone levels in amniotic fluid (*F*_1, 308_ = 86.0, *p* = 0.36).

## Discussion

This is the first study to demonstrate that an epidemiologically validated risk factor for ASD alters steroidogenesis in the foetal brain. The findings from this study have uncovered brain-related mechanisms that not only help to explain how DVD-deficiency may increase ASD risk, but may have broader implications for many of the other non-genetic risk modifying factors for ASD.

First, we show vitamin D deficiency significantly increased maternal testosterone levels. The hypothesis that maternal testosterone may be associated with ASD has several bodies of supportive evidence [39–42]. The incidence of ASD is higher in the children of mothers with testosterone-related medical conditions [40]. For instance, a remarkably consistent finding is that polycystic ovarian syndrome (PCOS) and preeclamptic women exhibit significantly higher levels of testosterone in their blood [41, 42]. Aromatase levels are frequently reported to be decreased in both these maternal conditions along with increased circulating testosterone specifically when the foetus is male [43, 44]. PCOS and preeclamptic women also have more than 50% increased odds of having a first child with ASD [45–47]. Interestingly, a randomised clinical trial showed that vitamin D supplementation reduced androstenedione levels in PCOS patients [48]. Vitamin D supplementation has also been shown to reduce testosterone in animal models of PCOS [49]. Despite these findings, to the best of our knowledge, there have been no studies examining the mechanism/s behind how vitamin D deficiency could lead to alterations in maternal testosterone levels.

In contrast to clinical studies, with animal models we can directly assess steroid levels in developing brains as well as underlying mechanism/s of abnormal steroidogenic activity locally in the foetal brain. We found that DVD-deficient embryos had small but significant elevation of testosterone in their brain which was statistically significant in males. Consistent with this we show DVD-deficiency produced a significant reduction in brain aromatase which is the principal catabolic enzyme in the elimination of testosterone [50]. We next explored the methylation of the aromatase promoter as one plausible mechanism [51–53]. We show one of the two brain aromatase promoters PII is hyper-methylated in male DVD-deficient foetal brains. Taken together hypermethylation of an aromatase promoter may silence its production leading to decreased testosterone elimination resulting in the increase in testosterone in DVD-deficient male brains.

**Fig. 5:**
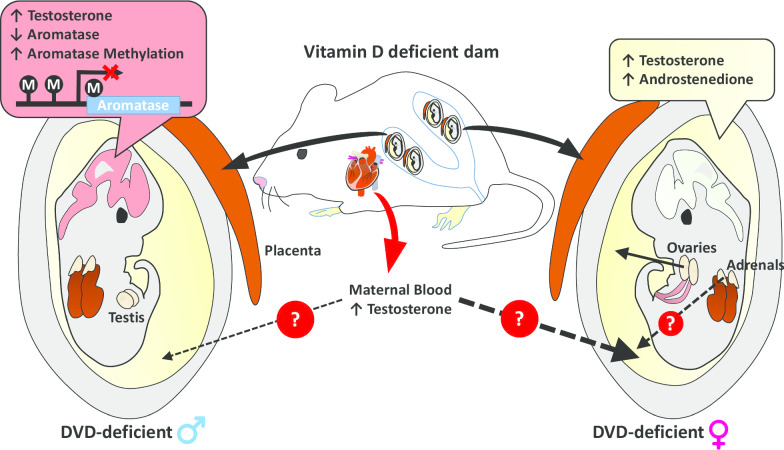
An illustration of the potential molecular mechanism and pathways involved in the exposure of developing foetus to increased testosterone. DVD-deficiency downregulates aromatase expression in male brains by increasing aromatase promoter methylation potentially increasing testosterone levels in male foetal brains. We further found that both testosterone and androstenedione were elevated in DVD-deficient female amniotic fluid which may be due to sex-specific effect of DVD-deficiency on androgen production in female embryonic ovaries or adrenal glands or a greater contribution from the increased testosterone levels in DVD-deficient dams. Dashed arrow represents hypothetical relevance of testosterone transfer

It is not ethically feasible to measure brain testosterone concentrations in human foetuses. However, the use of stored amniotic fluid can be considered a proxy marker of the combined outcome of foetal/placental steroid production. Whether maternal testosterone diffuses into the amniotic fluid has not yet been established [54]. Moreover, during late gestation, amniotic fluid reflects foetal urine [55]. Therefore amniotic fluid testosterone levels may be completely independent of maternal levels. In the past, amniotic fluid was the sample of choice for prenatal screening of risk factors that directly affect foetal growth [56]. Indeed, of direct relevance to the current study, increased levels of several steroids including testosterone and androstenedione have been found in the amniotic fluid of children later diagnosed with ASD [19]. Our findings here first show robust sex differences in amniotic fluid testosterone levels. This at least confirms that the foetus is the main source of these sex hormones. Our results further indicate that there is a clear effect of DVD-deficiency in elevating testosterone levels in female amniotic fluid. Androstenedione levels were also elevated in DVD-deficient amniotic fluid but similar to testosterone, this dietary effect was mainly observed in female amniotic fluid. This may suggest that DVD-deficiency may have a sex-specific effect on androgen production in female embryonic ovaries or adrenals but not male testes or adrenal glands; however, this requires further investigation. Previous studies have also shown sex-specific effects of DVD-deficiency on placental functions [57, 58]. However as aromatase is not expressed in rodent placenta [59], we believe the placenta is not playing an active part in these findings for this particular preclinical model. Though disputed [54, 60], if maternal testosterone is capable of diffusing through the placenta to foetal circulation, the contribution from the maternal component is likely to be far greater in the female embryo as the levels of testosterone production are far lower in the female as evidenced by levels observed in amniotic fluid and brain. A summary of all findings from this study is shown in Fig. 5.

Several studies have associated increased foetal testosterone with certain autistic traits such as reduced eye contact, delayed language development and narrow range of interests [20, 22, 61]. It remains unknown how increased exposure to testosterone in utero could produce ASD like symptoms in children. Testosterone is key early factor and plays important role in the development of sexually dimorphic brain regions in human [62] and also has profound effects on several neuronal processes during brain development such as neurogenesis, migration and immune functions [63–65]. This remains an intense area of research.

### Limitations

Aromatase is a key enzyme in the biosynthesis of estradiol. A reduction in aromatase expression may also lead to a reduction in estradiol levels. More importantly, a recent study suggests that elevated levels of amniotic fluid estrogens are associated with ASD, with estradiol levels being the most significant predictor of ASD diagnosis [66]. Unfortunately, estradiol was not analysed here. The aromatase protein and enzyme activity were also not assessed because of unavailability of sufficient embryonic brain samples. Moreover, we could not calculate the relationship between steroids levels within one foetal compartment i.e. foetal brain and amniotic fluid as these samples were taken from two separate cohorts of animals. Vitamin D is also known to promote the transcription of aromatase in different cell types [27, 67]. Therefore an alternative hypothesis may be that in the developmental absence of vitamin D the unliganded vitamin D receptor may bind known vitamin D response elements within the aromatase promoters [68] attracting a variety of known transcriptional repressors to suppress aromatase expression as has been shown for several other genes [69, 70]; however, this gene regulatory mechanism was also not assessed.

Progesterone is another important early steroid and developing brain is certainly sensitive to maternal progesterone during critical periods of development [71]. Alterations in progesterone levels have also been associated with increased risk of ASD [19]. Although we did not measure progesterone levels in maternal serum, we examined the RNA expression of progesterone related enzymes in the foetal brains. Progesterone is produced by enzymatic conversion of 3β-hydroxysteroid dehydrogenase 1 (*HSD3β1*) from the precursor, pregnenolone. We examined the gene expressions of both progesterone producing enzyme *HSD3β1* and its major catabolic enzyme *CYP17A1*. These enzymes were not altered by DVD-deficiency suggesting progesterone levels are similarly unlikely to be altered by DVD-deficiency. Based on these results, we did not further investigate this pathway [72].

## Conclusions

Increased foetal exposure to testosterone leading to increased androgenisation of the foetal brain is frequently cited as a possible causative process for the pronounced sex-bias in autism [73, 74]. However, this remains a difficult hypothesis to test directly in human embryos. Preclinical models based on other epidemiologically validated risk factors for ASD such as maternal immune activation or prenatal exposure to valproate produce social and communication deficits and stereotyped behaviours, all phenotypes of relevance to ASD [75–77]. Remarkably these phenotypes frequently appear to be more pronounced in males. Here we have examined a preclinical model for another epidemiologically validated developmental risk factor for ASD, DVD-deficiency. In this study we have explored the known clinical and cellular links between vitamin D and the regulation of testosterone. We have shown DVD-deficiency elevates testosterone directly in the foetal brain. Increased methylation of the aromatase promoter is consistent with reduced aromatase transcription in DVD-deficient foetal brains. Whether other epigenetic mechanisms are also operating such as regional alterations to histone acetylation/methylation in the foetal brain are currently unknown. We speculate the male bias in other preclinical models may also be due to such mechanisms. The Simons foundation autism research initiative has made understanding sex differences in ASD one of its top priorities for funding in 2020. The findings from this study should prompt the examination of other developmental risk factors for ASD and whether they produce male-selective outcomes via a similar mechanism.

## Supplementary information

**Additional file 1**: 1. Tissue collection. 2. 25OHD levels in the dams. **Supplementary Table 1**. 2-Methoxy-estradiol (2-ME) levels in the dams. 3. Foetal crown-rump length. 4. VDR expression in foetal brain. 5. Methylation status of aromatase PI.f promoter. **Supplementary Table 2**. Primer sequences used in MeDIP-qPCR experiments. 6. RNA extraction and qPCR conditions. **Supplementary Table 3**. Primer sequence information used in qPCR experiments. **Supplementary Table 4**. All possible combination of pairwise correlations between steroid levels and Aromatase.

## Data Availability

All data generated or analysed during this study are included in this published article and its supplementary information files.

## Abbreviations

ASD: Autism spectrum disorder
DVD: Developmental vitamin D
25OHD: Vitamin D
1,25OHD: 1,25-Dihydroxy-vitamin D
SD: Sprague–Dawley
E: Embryonic day
LC–MS/MS: Liquid chromatography/tandem mass spectrometry
MeDIP: Methylated DNA immunoprecipitation
*CYP19A1*: Aromatase
5mC: 5 Methylated cytosine
PII: Proximal promoter
PI.f: Brain promoter
PCOS: Polycystic ovarian syndrome
2-ME: 2-Methoxyestradiol
