# Supplementary material for: Developmental vitamin D deficiency increases foetal exposure to testosterone
Source: Mol Autism. 2020 Dec 10;11:96. doi: 10.1186/s13229-020-00399-2 (PMC7727109; doi:10.1186/s13229-020-00399-2)
Supplement: Supplementary file 1 — Additional file 1: 1. Tissue collection. 2. 25OHD levels in the dams. Supplementary Table 1. 2-Methoxy-estradiol (2-ME) levels in the dams. 3. Foetal crown-rump length. 4. VDR expression in foetal brain. 5. Methylation status of aromatase PI.f promoter. Supplementary Table 2. Primer sequences used in MeDIP-qPCR experiments. 6. RNA extraction and qPCR conditions. Supplementary Table 3. Primer sequence information used in qPCR experiments. Supplementary Table 4. All possible combination of pairwise correlations between steroid levels and Aromatase. [file 13229_2020_399_MOESM1_ESM.docx]

**Supplementary data**

**1. Tissue collection**

Each foetus was removed from the uterus with the amniotic sac intact. Amniotic sacs were punctured in a sterile petri dish to collect amniotic fluid which was snap-frozen in liquid nitrogen at -80 °C. The crown-rump length was measured from each foetus prior to decapitation to collect foetal brains. Foetal tails were collected for the identification of foetal sex by amplification of sex-determining region Y gene (1). Maternal vitamin D deficiency was confirmed by measuring sera 25OHD levels from maternal blood. All samples were collected between 10:00 and 13:00 h to minimize diurnal variation. The whole study was conducted in two different cohorts. To account for litter effects and maximise the biological variance, a minimum of 12 litters from each diet group were used in each cohort. For quantification of steroid levels from foetal brains, a total of 318 (150 control + 168 DVD-deficient) foetal brains were collected from cohort 1 dams, and all of them were used in this experiment. RNA expression and DNA methylation were conducted on cohort 2 animals. For RNA expression, a total of 45 foetal brain samples [24 controls (2 per litter) + 21 DVD-deficient (2 per litter)] were used. However, for DNA methylation, 4 controls and 4 DVD-deficient foetal brains were used collected from 4 separate litters of each diet group. Steroid levels from amniotic fluid were also measured from cohort 2. A total of 308 (166 control + 142 DVD-deficient) samples were collected from 24 litters and all of them were used in this experiment. See figure legends for more details. Sex steroids were measured from the dam’s blood samples from both cohorts. See figure legends in the manuscript for more details.

**2. 25OHD levels in the dams**

As expected DVD-deficient dams had significantly (F_1, 28_ = 93.22, p < 0.001) lower serum 25OHD levels compared to dams on the control diet [(control=28.33 nmol/L ±2.9, DVD-deficient=0.97nmol/L ±0.28 (± standard error mean)].

**Supplementary Table 1**

**2-Methoxy-estradiol (2-ME) levels in the dams**2-Methoxy-estradiol (2-ME) levels were also measured in the dam’s sera and shown to be unaffected by DVD-deficiency.

| **Diet Group** | **Mean levels (pg/mL)** | **Std. Deviation** | **n** |
| --- | --- | --- | --- |
| Control | 380.6769 | 38.31634 | 13 |
| Vitamin D deficient | 370.1923 | 18.49006 | 13 |

**3. Foetal crown-rump length**

The mean foetal crown-rump length did not vary between experimental groups; [(control=21.85 mm±0.213, DVD-deficient=21.37mm±0.208 (± standard error mean)], suggesting no effect of DVD-deficiency on gross foetal morphology.

**4. VDR expression in foetal brain**

The expression of the VDR in the foetal brain (E18) was not significantly different between control and DVD-deficient groups (t_(43)_= 1.75, p = 0.64).

*Supplementary Figure 1. Expression of vitamin D receptor (VDR) in the foetal brain at E18.*

**5. Methylation status of aromatase PI.f promoter**

There were no significant differences observed for the PI.f promoter methylation (t_(7)_ = 1.65, p = 0.14) between DVD-deficient and control foetal brains.

*Supplementary Figure 2. 5mC levels within PI.f promoter in the male foetal brains*

**Supplementary Table 2**

**Primer sequences used in MeDIP-qPCR experiments**

| **Gene** | **Accession no** | **Sequence** | **Genomic region**  **(bp from TSS)** | **Product length** |
| --- | --- | --- | --- | --- |
| ***PII 1*** | NM_017085.2 | CCCCAAGCTAGTTTTCTTTGTGTG | *-1688 to -1529* | 160 |
|  |  | GTGAGTTCGAGGCAAACCTG |  |  |
| ***PII 2*** |  | AGTTCCAGGGCTCAAGTTCA | *-1208 to -1026* | 183 |
|  |  | AAGTGGAGGGTGAAGGGTCT |  |  |
| ***PII 3*** |  | TCTGTGGTTGCATGAAGGTC | *-892 to -742* | 151 |
|  |  | ATTGTGTAAGCCGCCAGAAC |  |  |
| ***PII 4*** |  | CCAGCGTCTTCAACTTGGTT | *-392 to -235* | 158 |
|  |  | AGCGACTCAGGTGTCACTCA |  |  |
| ***PII 5*** |  | CAAGGGTAGGAATTGGGACATTG | *74 to 225* | 152 |
|  |  | ATCCCCTAGGACACACATGC |  |  |
| ***PI.f 1*** | EF474097.1 | CTTAAATGCTGTGCCCATTCATAG | *-37853 to -37685* | 169 |
|  |  | ACAGCACACAATGGGTTTGA |  |  |
| ***PI.f 2*** |  | TCTTCATCCCCAGTGTAGGCT | *-37613 to -37380* | 234 |
|  |  | GGAGGAGGAGGAGGAGAAGAAG |  |  |
| ***PI.f 3*** |  | CTTGTGATTAGCCCCTTTTTGCC | *-37057 to -36867* | 190 |
|  |  | TGCCCAAAAAGAAACTGTCCCTC |  |  |
| ***PI.f 4*** |  | ACCACAGAGAGTGAAAGTTTGAG | *-36374 to -36214* | 160 |
|  |  | GCGTACCAAGAGAAGCCAAT |  |  |

**6. RNA extraction and qPCR conditions**

Total RNA was extracted from the whole foetal brain using RNeasy Mini Kit (Cat No. 74104) Qiagen and 5 μg of RNA was reverse transcribed into cDNA by using SuperScript IV First-Strand Synthesis System (Cat No. 18091150) Invitrogen. qPCR was performed in duplicate in 12 μl reaction mixtures using 480 SYBR Green I Master Mix (Cat No. 04707516001) by Roche Diagnostics, Penzberg, Germany. The reaction was performed in a LightCycler® 480 System (Roche Diagnostics, Penzberg Germany) under following conditions: initial denaturation at 95 °C for 5 min followed by 40 cycles of amplification (95 °C for 10 s, then 60 °C for 20 s, then 72 °C for 20 s). The relative expression of the genes examined was normalized to that of endogenous control glyceraldehyde 3-phosphate dehydrogenase (GAPDH).

**Supplementary Table 3**

**Primer sequence information used in qPCR experiments**

| **Gene** | **Accession no** | **Sequence** | **Product length** |
| --- | --- | --- | --- |
| **Aromatase** | NM_017085.2 | CTCCTCCTGATTCGGAATTGT | 90 |
|  |  | TCTGCCATGGGAAATGAGAG |  |
| **Cyp21a1** | NM_057101.2 | GACATGATTGACTACATGCTCCAG | 165 |
|  |  | GTGAAGCAGGAAAGCCACAG |  |
| **Cyp11a1** | NM_017286.2 | CAACATCACAGAGATGCTGGCAGG | 559 |
|  |  | CTCAGGCATCAGGATGAGGTTGAA |  |
| **Cyp11b1** | NM_012537.3 | TTGCTAAGGACTGGGTGGTTGT | 237 |
|  |  | AACTTTTCGCCCTACCGACTTG |  |
| **Cyp17a1** | XM_006231435.2 | GCAGAGGTTTGACTTGGATGTG | 180 |
|  |  | GAGGTATGGATCGGGGATGTTA |  |
| **Hsd3β1** | NM_001007719.3 | CCCTGCTCTACTGGCTTGC | 189 |
|  |  | TCTGCTTGGCTTCCTCCC |  |
| **Hsd11β2** | XM_006255445.2 | CTCTTGAAATGAAGCAGGGCC | 200 |
|  |  | CTCTTGCTCAGGTTCAAGACC |  |
| **Hsd17β3** | NM_054007.1 | CTCCCCAACCTGCTCCCAAGTCATTT | 408 |
|  |  | AGCAAGGCAGCCACAGGTTTCAGC |  |
| **HPRT** | XM_008773659.2 | TTCTTTGCTGACCTGCTGGA | 117 |
|  |  | CCCCGTTGACTGGTCATTACA |  |
| **Srd5a1** | NM_017070.3 | ACCTTTGTCTTGGCCTTCCT | 490 |
|  |  | GGTCACCCAGTCTTCAGCAT |  |

**Supplementary Table 4**

All possible combination of pairwise correlations between steroid levels and Aromatase

| **Correlation** | **Pearson r** | **R squared** | **N** | **Sig. (2-tailed)** |
| --- | --- | --- | --- | --- |
| **Aromatase gene expression vs Aromatase PII MeDIP** | 0.285 | 0.081 | 40 | 0.075 |
| **Aromatase gene expression vs Aromatase PI.f MeDIP** | 0.093 | 0.009 | 32 | 0.614 |
| **Foetal Brain TEST (m) vs maternal TEST** | 0.140 | 0.002 | 30 | 0.459 |
| **Foetal Brain TEST (f) vs maternal TEST** | 0.292 | 0.085 | 30 | 0.117 |
| **Foetal Brain AND (m) vs maternal AND** | 0.141 | 0.002 | 30 | 0.828 |
| **Foetal Brain AND (f) vs maternal AND** | 0.087 | 0.008 | 30 | 0.648 |
| **Foetal Brain CORT (m) vs maternal CORT** | 0.053 | 0.003 | 30 | 0.780 |
| **Foetal Brain CORT (f) vs maternal CORT** | 0.090 | 0.008 | 30 | 0.635 |
| **Amniotic fluid TEST (m) vs maternal TEST** | 0.097 | 0.009 | 32 | 0.597 |
| **Amniotic fluid TEST (f) vs maternal TEST** | 0.037 | 0.003 | 32 | 0.839 |
| **Amniotic fluid AND (m) vs maternal AND** | 0.180 | 0.033 | 32 | 0.323 |
| **Amniotic fluid AND (f) vs maternal AND** | 0.061 | 0.008 | 32 | 0.743 |
| **Amniotic fluid CORT (m) vs maternal CORT** | 0.457 | 0.209 | 32 | 0.009* |
| **Amniotic fluid CORT (f) vs maternal CORT** | 0.132 | 0.018 | 32 | 0.472 |

Statistically a significant correlation was found between corticosterone levels in male amniotic fluid and dam’s blood. No significant correlation were evident for any other association. m=males, f=females, TEST=Testosterone, AND=androstenedione, CORT=corticosterone, *> P 0.01.

**References**

1. Sathishkumar K, Elkins R, Chinnathambi V, Gao H, Hankins GD, Yallampalli C. Prenatal testosterone-induced fetal growth restriction is associated with down-regulation of rat placental amino acid transport. Reproductive biology and endocrinology : RB&E. 2011;9:110.

2. Hu S, Wan J, Su Y, Song Q, Zeng Y, Nguyen HN, et al. DNA methylation presents distinct binding sites for human transcription factors. Elife. 2013;2:e00726.
